# Emerging Trends of Ergonomic Risk Assessment in Construction Safety Management: A Scientometric Visualization Analysis

**DOI:** 10.3390/ijerph192316120

**Published:** 2022-12-02

**Authors:** Rakhi Vijayakumar, Jae-ho Choi

**Affiliations:** ICT Integrated Safety Ocean Smart Cities Engineering Department, Dong-A University, S12-401, 550 Bungil 37, Nakdong-daero, Saha-gu, Busan 49315, Republic of Korea

**Keywords:** construction, ergonomic risk assessment, occupational safety and health, scientometric analysis, VOSviewer, work-related musculoskeletal disorder

## Abstract

Ergonomic risk assessment is critical for identifying working posture hazardous to the health of construction workers. Work-related musculoskeletal disorders (WMSDs) are predominant non-fatal injuries in the construction industry owing to manual handling activities and poor working conditions. However, there is a lack of scientific synopsis aiming to better understand the emerging research focus in this field. To fill the research gap, this study performed a scientometric evaluation of the bibliometric data on ergonomic risk assessment from the Web of Science database using VOSviewer software. The purpose of this study is to analyze the co-occurrence network of keywords, co-authorship network, most active countries, and the sources of publication. The results indicate that research related to risk assessment in construction has fluctuating growth, peaking in 2020 with significant advancements in the USA, China, and Canada. WMSDs, risk factors, construction workers, and ergonomics are hot research topics in this field. Furthermore, the research gaps of previous studies and suggestions for future research have been provided to bridge the knowledge gap. We believe that this scientometric review provides useful reference points for early-stage researchers as well as beneficial in-depth information to experienced practitioners and scholars in the construction industry.

## 1. Introduction

Construction projects are increasingly emerging as difficult and complex owing to high-risk activities. Consequently, construction professionals need a wide range of abilities to be capable of completing projects on schedule, within budget, and with the highest quality [1,2]. Worker activities in the construction industry are typically repetitive and physically strenuous. Performing such tasks in awkward positions can strain workers’ bodies, resulting in fatigue, injury, or in severe cases, permanent disability [3,4,5]. Work-related musculoskeletal disorders (WMSDs) are the primary contributing factor to non-fatal injuries in construction, accounting for 27.5% of non-fatal construction injuries in the United States and 18% in Hong Kong [6]. Common risk factors of WMSD include repetitive motion, high-force exertion, awkward body posture, vibration, and contact force [7,8,9]. Additionally, WMSDs are linked to high employer expenses due to absenteeism, decreased productivity, higher healthcare costs, and workers’ compensation [6,10,11]. Therefore, proper management of employee safety and health is critical not only in creating a better working environment, but also for contractors’ direct and indirect accident costs.

Ergonomics refers to the study of designing a safe and productive workplace, work habits, and workflows for workers in order to eliminate risk factors including sprains, strains, and cumulative trauma disorders, and to assess the risk level for tasks related to work. It analyzes people’s behavioral, psychological, and physiological capacities and restrictions [9,12]. Previous studies have used several ergonomic risk assessment tools such as the Posture Activity Tools Handling (PATH), Ovako Working posture Analysis System (OWAS), Rapid Entire Body Assessment (REBA), and the Rapid Upper Limb Assessment (RULA), and so on. These tools enabled the evaluation of the risk associated with certain activities by simulating tasks in the laboratory environment and also by onsite evaluation [13,14,15,16].

In the last decade, construction workers’ safety was given more significance as more diverse emerging technologies were integrated into construction-related projects [17]. A literature review is thought to be a quick and easy way to comprehend a research area thoroughly [18]. Due to the immense use of ergonomic tools in reducing injuries among construction workers, literature related to this domain has greatly expanded, and several literature reviews on ergonomics in construction have been presented. Some studies concentrated on reviewing the prevalence of various risk factors and their association with WMSDs in construction. For instance, Kittuswamy and Buchholz [19] provided a review of the literature that studied the exposure of mobile equipment operators to two important health hazards: whole-body vibration and postural stress. Another study by Sobeih et al. [20] critically evaluated the epidemiological research relating psychosocial work variables to musculoskeletal diseases in construction workers. A definition of ergonomics and an overview of risk factors is presented in a review by Jaffar et al. [12]. Anagha and Annie [21] reviewed several ergonomic risk factors causing WMSDs in construction. A more specific review of the connection between physical and psychosocial risk factors and WMSD in different construction trades is investigated by Anwer et al. [22].

The concept of ergonomics, its definitions, history, and its integration in the Malaysian construction industry are highlighted in a paper by Tharim et al. [23], although they did not overview the state-of-the-art ergonomic risk assessment practices. Currently, only a limited number of literature reviews focus on identifying existing ergonomic risk assessment methods in the construction industry. Inyang et al. [24] reviewed state-of-the-art ergonomic techniques and the occupational health and safety impact of WMSDs resulting from existing practice and legislation. Wang et al. [7] reviewed the available methods for WMSD risk assessment and highlighted their advantages and drawbacks. The data provided in their work is limited by timeliness, and computer-vision-based technologies are not explained adequately, downscaling their importance in ergonomics. Valero et al. [25] presented an in-depth review of the postural assessment methods focusing specifically on inertial measurement units (IMUs). However, it is restricted to only one specific technique of ergonomic assessment which limits its potential in broadening the knowledge domain of readers. Moreover, the existing reviews discussed are manual and the results rely on subjective judgment.

Despite the importance of occupational health and safety among construction workers, we can conclude that there is a lack of an in-depth and sophisticated review that fully explores the evolutionary trends of research in the ergonomics domain in the construction industry. To overcome and expand on the shortcomings of previous literature, this study used a scientometric analysis to grasp what research has been produced so far, as well as the advancing trends and the fundamental research gaps. This study therefore serves to enhance the risk assessment practices in the construction industry. Our study effort is to:

1. Determine global hot topics and research trends in the construction ergonomic risk assessment;
2. Summarize the state-of-the-art developments in the ergonomics domain of construction;
3. Analyze the academic collaboration between authors;
4. Recommend future research directions in the domain.

This study is believed to be significant for academic researchers and practitioners to apprehend and perceive the emerging key topics associated with ergonomic risk assessment in construction. The remainder of the paper is organized as follows. Section 2 describes the bibliometric and scientometric methods used in this study. The visualization maps including co-author, co-occurring keywords, and citation analysis are discussed in Section 3. Section 4 describes the discussion, and the conclusions are provided in Section 5.

## 2. Materials and Methods

**Figure 1:**
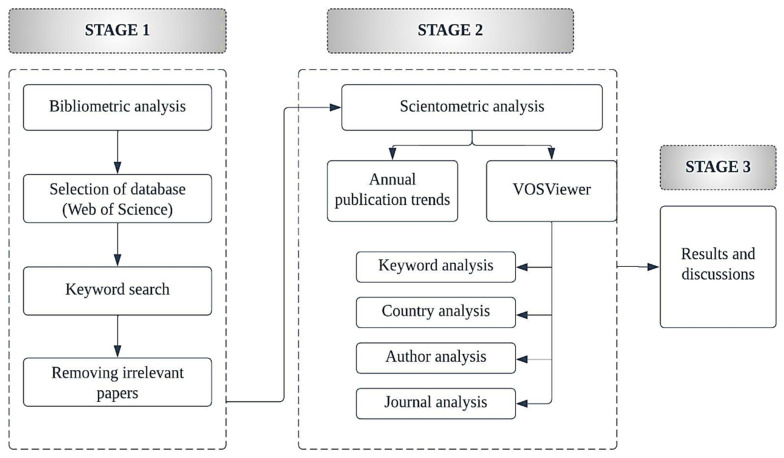
The overall methodology of three-stage scientometric analysis.

A range of methodologies is available in order to review the publications, out of which scientometric analysis excels at highlighting notable patterns and trends based on the author, keyword, and reference in a substantial body of literature data [26]. Therefore, a literature review based on the science mapping three-stage approach is applied in this study. The authors conducted a bibliometric analysis utilizing the Web of Science (WoS) database and a scientometric analysis using VOSviewer. The three stages include bibliometric analysis, scientometric analysis, and results and discussion. The overall methodology conducted in the review process is presented in Figure 1.

### 2.1. Bibliometric Analysis

The bibliometric analysis employs a statistical analysis using descriptive and evaluative approaches to display or capture publishing characteristics and research trends [27,28]. It has been widely used in literature reviews to present general features of a scientific problem including authors, journals, institutions, and keywords, etc. [29]. The first stage of research commenced with a comprehensive literature search conducted in the WoS database to retrieve publications related to ergonomics risk assessment in the construction industry in October 2022. The retrieval period of the journal article is restricted between 2000 and 2022 to provide a summary of the research advancement over the previous 23 years.

The authors used “TS = Work-related musculoskeletal disorders” OR “Musculoskeletal disorders” OR “Ergonomic* risk assessment” OR “Ergonomic tools” OR “Awkward posture” AND “TS = Construction” (with TS = topic search) as the search statement to conduct a literature query which initially yielded 413 results. This provided all necessary bibliographic details, including the title, author, abstract, keywords, sources, and references. The research was further refined to include only articles and conference proceedings, thus resulting in 379 publications. English was the prerequisite language specified for selecting articles. Finally, manual screening was performed to exclude literature not pertaining to the topic and 330 documents were obtained, which were then used in the scientometric analysis.

### 2.2. Scientometric Analysis 

The scientometric analysis provides a quantitative examination of all aspects of scientific literature in order to detect knowledge structure, scientific contributions, research advancements, and new trends in the study domain [30]. The purpose of the scientometric analysis is to generate institutional productivity comparisons, institutional research rankings, and journal rankings assessing top research articles, and create profiles of leading authors and institutions regarding research performance [31]. Text mining and citation analysis are two components of scientometric analysis, which assist researchers in identifying systematic literature-related insights by identifying literature data that might be overlooked in a manual review [26]. Several tools are available to researchers for performing scientometric analysis such as Citespace, VOSviewer, and Gephi. The large range of visualization options in the user interface of Citespace makes it difficult to understand and takes more time to learn, whereas, in Gephi, there is no particular facility for processing bibliographic data, necessitating the use of additional software tools. VOSviewer can create powerful graphical visualization and can work with large-scale data [32]. Hence, visualization of networks and citation analysis was conducted in this study using VOSviewer. 

The VOSviewer is an easy-to-use tool that can be used to create maps of authors or journals based on data from co-citations or maps of keywords based on data from co-occurrences [33]. In addition to having unique text-mining features, VOSviewer provides the fundamental functionality needed for creating, visualizing, and understanding bibliometric networks [26]. VOSviewer was used in this study to visualize keyword co-occurrence analysis, country co-authorship analysis, author analysis, and journal citation analysis. The 330 publications extracted after the final screening were downloaded from WoS as a tab-delimited file which was then imported into the VOSviewer software (version: 1.6.18). Visualizations can be generated and displayed in different views in VOSviewer such as network, overlay, and density visualization. Network analysis was used in this study in which the trends and changes in the main research areas and the hot topics were identified first using the keyword co-occurrence network. Secondly, the actively participating countries/regions in this field were determined by applying country analysis. Through the co-authorship network, the research experts in the field of ergonomic risk assessment and their relationships were assessed. Citation analysis was performed to identify the most productive journals.

## 3. Results of Scientometric Analysis

### 3.1. Annual Publication Trend

**Figure 2:**
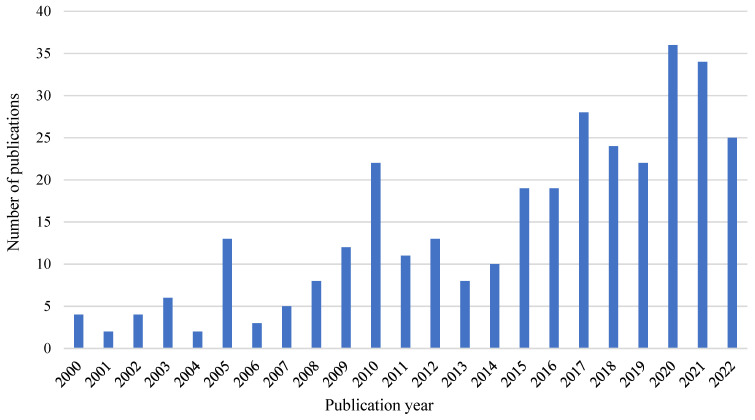
Annual publication trends of articles from 2000 to 2022 (October).

In general, the number of publications can be used to measure research activity, examine trends in the subject’s growth, and forecast future directions for research advancement [34]. Figure 2 indicates the annual publication trend on ergonomic risk assessment in construction. The evolution of publications can be divided into three stages. The first stage, from 2000 to 2005, encountered a slow discontinuous growth with a minimum publication of two every year. In this stage, a rapid increase in the publication was recorded from 2004 to 2005 with the number rising from 2 to 13 papers. The second phase noted a steady growth from 3 papers in 2006 to 22 papers in 2010. The third stage, between 2011 and 2020, recorded 190 works of literature with several fluctuations in 2013, 2018, and 2019, which is a significant growth compared to the other two phases. This indicates that the importance of ergonomics risk management gained attention among researchers during this phase. In the current year, the number of publications in this area of research is 25 thus far (October 2022). The focus of researchers on the importance of ergonomic risk assessment in construction has increased remarkably over the years.

### 3.2. Keyword Analysis

**Figure 3:**
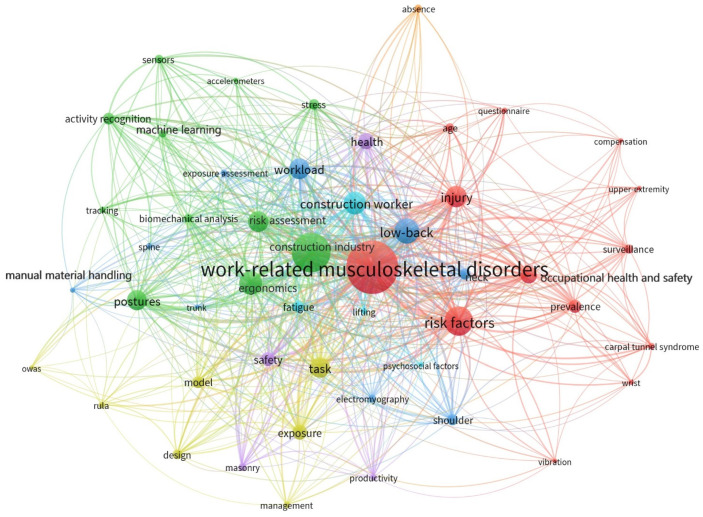
Co-occurrence of keywords.

In general, the number of times certain keywords appear together in a document is known as co-occurrence [35]. Co-occurring keyword network analysis gives researchers access to the fundamental data of core research material and enables them to examine the trends in the development of research subjects at various phases of ergonomic risk assessment studies. An effective way to understand a knowledge domain is through a network of keywords, which can be used to identify the interests over a particular period and demonstrate how they are connected and structured [26]. The network shown in Figure 3 indicates the hot topics in the ergonomics domain. 

The visualization and mapping of the network indicating the ergonomics knowledge domain in construction were obtained using VOSviewer. “Co-occurrence keywords” and “All keywords” were used in this study. The full counting option is used to run the co-occurrence analysis. According to the examination, ergonomic risk assessment in construction research papers included 1493 keywords. To refine the findings, a threshold of 5 keyword occurrences was applied, in which only 134 keywords met this requirement. Identical terms, such as “musculoskeletal disorders” and “work-related musculoskeletal disorders”, and “construction industry” and “construction sectors” were combined to generate the terms “work-related musculoskeletal disorders” and “construction industry”, respectively. The resulting network included 49 nodes and 631 relations which are grouped into 7 clusters, as displayed in Figure 3. 

Each node represents keywords, and the size of the nodes reflects the number of documents associated with each term [36]. The weighting increases as nodes (circles) get larger and the strength increases as the distance decreases [28]. The occurrences show how many articles included the keyword. Additionally, links refer to the co-occurrence of one entity (such as a keyword) with another, and the overall link strength relates to all the references that have been recognized between that entity and the others [37]. The two most occurring keywords in the topic study are WMSDs and the construction industry. WMSDs constitute the major occupational health and safety issue in the construction industry [38,39]. To indicate the seven clusters, seven different colors are used which indicate the different knowledge areas. 

#### 3.2.1. Ergonomics and WMSDs in Construction

From the network in Figure 3, it can be seen that the keyword “WMSD” which has a highest occurrence rate, has a strong link with “ergonomics” and “construction workers”. WMSDs are the most common ergonomics hazard prevalent in the construction industry. The most common types of WMSDS are Carpal tunnel syndrome, Tendonitis, and Bursitis [2]. Several studies evaluated the prevalence of WMSDs among construction workers. A study conducted by Algadhir and Anwer [40] in Saudi Arabia demonstrated that 48.5% of workers had musculoskeletal pain. Similarly, the 12-month prevalence rate in Nigeria and southeast Ethiopia showed 66% and 43.9%, respectively [41,42].

#### 3.2.2. Postural Risk Assessment in Ergonomics

The keyword “postures” has a strong association with the keywords “ergonomics”, “risk factors”, “construction industry”, and “construction workers”. Awkward postures are one of the main reasons of major health concerns in construction workers [43]. Over the years, scientists used observational methods like Rapid Entire Body Assessment (REBA), Rapid Upper Limb Assessment (RULA), Ovako Working Posture Analysis System (OWAS), and Posture, Activity, Tools, Handling (PATH), etc., to determine unsafe postures of workers. Ergonomic risk assessment of infrastructure construction workers in Kuala Lumpur was done using a Nordic questionnaire and REBA [44]. They selected bar benders, carpenters, and general workers for this study which highlighted that the lower back is the body part with the most supreme pain and discomfort. Furthermore, RULA is an upper body observational tool which can be used to identify ergonomic issues during manual handling tasks and plastering works. Mungroo and Choi [45] investigated WMSD risk factors among roofers in Central Trinidad. The results demonstrated that the highest risk occurred during sheet installation and the back and shoulders were most affected due to over-exertion. Similarly, PATH provides specialty-task activity estimates of hazardous construction ironwork, drywall workers, and residential building workers. This can be applied in assessing the risk associated with manual material handling, drywall panel work, and iron work [14,46,47].

Although these methods are easy to use, they require a trained observer which can impose additional costs. The results obtained may not be reliable since they depend upon the subjective assessment of the observer which can vary depending on the observer’s knowledge, leading to bias. Also, these methods require on-site observations or questionnaires to collect postural data, hence they can interrupt the activities of workers. As the construction sites are large and various activities will be ongoing, it can be time-consuming to observe each activity separately. This led to the utilization of sensors in postural data collection. For instance, Valero et al. [8] used IMUs to identify awkward postures in brick-laying activity. The major drawback of applying sensors in the estimation of construction workers’ posture is the intrusiveness caused by body-attached sensors. This was overcome by the advent of computer vision and several studies integrated deep learning-based approaches in the observation-based risk assessment of construction workers to obtain postural data. Chu et al. [48] developed a monocular vision-based biomechanical analysis model for modular construction. An open-source computer vision technology, OpenPose, combined with REBA or RULA for ergonomic postural assessment, has been proposed in several studies [49].

#### 3.2.3. Risk Factors of WMSDs and the Most Affected Body Parts

The first cluster (red) and third cluster (blue) outline the prevalence of risk factors in the construction industry. Construction workers are exposed to various risk factors which can be categorized into three: physical (biomechanical), psychosocial, and individual risk factors. Physical risk factors are dangerous activities or conditions that expose employees at risk for WMSD. They entail repetitive movements, heavy lifting, awkward postures, and poor working environments such as those with high vibrations and extreme temperatures. Social problems including family conflicts, safety concerns, and time constraints can be sources of psychosocial pressures. Gender, age, poor physical or mental health, and bad habits, constitute individual risk factors and can differ from person to person [7].

According to the framework, the body parts most affected by musculoskeletal injury are the shoulders, low-back, neck, spine, and trunk. The study by Dong et al. [50] in 2017 showed that the predominant body parts affected by WMSDs which resulted in days away from work were the back (41.7%) and shoulders (12%). Back disorders can be prominently found in construction activities, specifically in masonry workers, rebar workers, roofing workers, and framing carpenters. Surface electromyography (sEMG) and Kinect sensors can be used to evaluate back injuries among workers [51,52,53].

#### 3.2.4. Ergonomics and Construction Subsectors

The prevalence of WMSDs among workers varies with different sub-activities of the construction industry. Among various construction activities, manual material handling and masonry have gained popularity over the years. This could be due to the high prevalence rate of musculoskeletal symptoms while executing this task. The keyword “masonry” shows a connection with “electromyography” which indicates that this technique is used to evaluate the postures and workload involved in this task. A study by Anton et al. [54] evaluated the presence of musculoskeletal symptoms among brick and block apprentices using self-reported surveys. Another survey found that the most affected areas of masonry workers are hand/wrist, followed by knee/leg and back [55]. Furthermore, one of the riskiest tasks in construction is manual material handling, which includes pushing and pulling, and lifting and lowering goods [56].

The research by Lipscomb et al. [57] indicated that the majority of back injuries among carpenters are caused by overexertion injuries from manual material handling activities. An sEMG was used to assess the muscle activity developed during manual lifting tasks in construction, and risk assessment was carried out using the NIOSH lifting equation [58]. Another study developed a passive exoskeleton to measure spinal biomechanics during manual material handling using sEMG [59]. Additionally, a vision-based method to classify the manual handling tasks for ergonomic assessment based on OWAS was proposed by [6]. The findings will benefit aspiring researchers in determining keywords that make it easier to find literature that has been published on a particular subject.

### 3.3. Country Analysis

**Figure 4:**
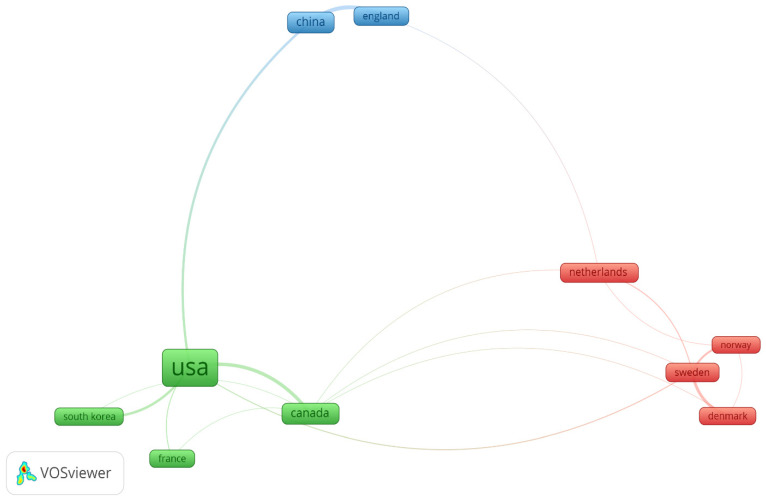
Visualization of co-authorship by country.

The co-authorship analysis makes it possible to visualize the patterns of collaboration between or among organizations and individuals [28]. In this study, co-authorship analysis based on countries was adopted to analyze geographical collaborations. To run by country, a threshold of a minimum number of documents and a minimum number of citations for a country was set as 10, and out of the 46 countries, 10 satisfied it. Figure 4 depicts the visualization and Table 1 presents the number of publications per country, the total number of citations they have had, and the total strength of the links in connection with co-authorship. The countries with more collaboration with each other were grouped into one cluster of the same color [60]. The placement of the authors in the Netherlands, Sweden, Denmark, and Norway together indicates that these researchers have close collaboration in the ergonomic assessment studies in the construction industry. 

The network consists of three clusters. The first cluster encompasses Denmark, Netherlands, Norway, and Sweden. The second cluster includes Canada, France, South Korea, and the USA. R The third cluster includes China and England. The USA stands out as the territory with the most published documents (131) compared to other countries, and also gained the highest citation of 2987. This indicates that the USA is the most productive and influential country in the topic of interest. The authors in Canada have more collaborative projects in ergonomics with other countries.

### 3.4. Author Analysis

**Figure 5:**
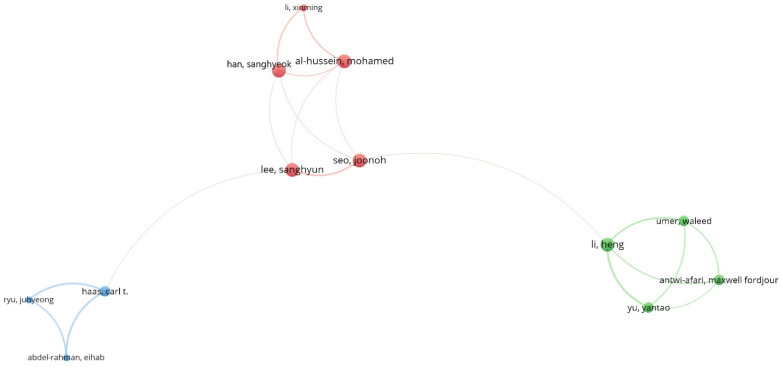
Systematic map of researchers.

Citations demonstrate an author’s contribution to a specific area of research [61,62,63]. Co-authorship is selected as the basis of analysis in which authors are chosen as the unit of analysis for the collaborative assessment of authors. To evaluate a researcher’s performance, total citations, total publications, and average citations are taken into account separately [63]. The minimum number of documents of an author was kept at 5, and 36 of the 1008 authors met the requirements. Table 2 lists the authors with the most papers and citations, and Figure 5 depicts the scientific mapping of researchers. The average citation count was calculated by dividing the total number of citations by the sum of all publications for each author [64]. From this network, it is evident that the collaboration between authors in this field can be divided into three clusters. The authors in the green cluster mainly focused on biomechanical analysis of risk factors using wearable insole sensor data and sEMG [65,66,67,68]. The leading researcher is Li with 14 publications, followed by Dale and Veiersted with 9 publications. In terms of citations, Li leads the field with 511 citations, followed by Seo with 182 citations in the ergonomics risk assessment research area. Furthermore, when the average citation is considered, the authors can be ranked as Li, Seo, and Lee with 36, 37, and 29 average citations, respectively. According to the study, it was revealed that 12 of the 36 authors had citations linking them to one another. Based on citations, it was discovered that numerous authors from various regions are connected in the area of ergonomic risk assessment in construction.

### 3.5. Journal Analysis

**Figure 6:**
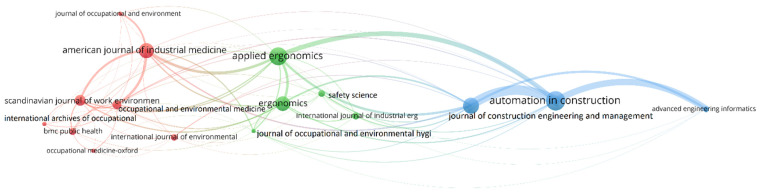
Source analysis.

The sources of ergonomics-related publications were identified using VOSviewer. Journals serve as a primary basis for several fields of topical research, and also provide information on dominant spaces and platforms for researchers to present their research findings for the best impact in academia and industry [35]. Figure 6 represents the network analysis of the most productive journals and Table 3 illustrates the detailed results of journal sources of risk assessment literature. For the analysis of sources, “citation” is selected as the type of analysis with “sources” as the unit of analysis. The “minimum number of documents of a source” and “minimum number of citations of a source” was specified as five. Consequently, 16 sources out of 119 fulfilled the threshold. In Figure 6, the bigger nodes indicate the journals with more articles published. The thicker lines indicate the sources with greater citations among them. “*Automation in construction*” has a better relationship with “*Journal of construction engineering and management*”, “*Advanced engineering informatics*”, and “*Applied ergonomics*”. As depicted in the table, “*Automation in construction”*, “*Applied ergonomics*”, and “*Journal of construction engineering and management*” are the top publishing journals with 26, 24, and 21 papers, respectively, indicating that these journals have a high influence in this field. However, the most cited journals are “*Automation in construction*,” “*American journal of industrial medicine*”, and *Occupational and environmental medicine*”.

## 4. Discussions

### 4.1. Benefits of Ergonomic Risk Assessment in Construction

The construction sector has the unique distinction of being one of the world’s most dangerous and injury-prone industries. It entails using machinery and power tools continuously, operating on high scaffolds, and manual handling, etc. [69]. The injuries and occupational health issues arising from poor working conditions have an influence on a country’s economy and the welfare of workers. Work-related musculoskeletal disorders are common occupational hazards resulting from exposure to unhealthy working conditions. According to a study by Dong et al. [50], the construction sector had 31.2% WMSD which required days away from work. Ergonomics ensures an efficient, secure, and comfortable workplace, and ergonomic methods have facilitated safe and healthy practices for construction workers to prevent fatalities. The safety and productivity of workers in a construction site are closely related to working postures. Several methods have been developed for the risk assessment of workers’ posture and unsafe behaviors. For instance, questionnaires were used as a tool to identify the risk factors persistent among various activities. Some observational tools like REBA and RULA are prevalent in estimating the risk associated with a particular activity indicated by risk score. Furthermore, wearable sensors like IMUs are also utilized to obtain the muscle activities of fatigue of workers. Additionally, computer-vision-based methods are incorporated into this area of research to achieve real-time performance. The keyword co-occurrence network emphasizes that researchers have recognized the advantages of implementing risk assessment models or techniques on the economy and occupational health and safety of a country.

### 4.2. Challenges and Future Directions

#### 4.2.1. Application in Construction Activities

According to keyword co-occurrence mapping, masonry and manual material handling received more attention in risk assessment. This can be attributed to the heavy lifting activities involved in masonry work and manual handling tasks that may lead to WMSDs. Jennifer A. Hess et al. [70] evaluated shoulder exposure and low back stress among masonry workers by capturing videos and assessments using observational tools. However, a recent study exhibited that the incident rate of WMSDs was found to be higher among tiling and painting workers, with incident rates corresponding to 125% and 75.2%, respectively, whereas masonry was 30.1% [50]. No study has evaluated the risk factors associated with these two activities according to the keyword, title, and abstract analysis. Future research work should include onsite or laboratory observation of tiling and painting activities to recognize unsafe behaviors to prevent fatalities. Additionally, the studies mostly concentrated on investigating the neck, shoulders, and low back stress. However, real-time full-body posture analysis can give more accurate results regarding the bad postures leading to WMSDs.

#### 4.2.2. Collaboration between Authors

The insufficient collaboration between authors reflects the lack of knowledge distribution and productivity in a specific field. Collaborative relationships are encouraged to broaden the knowledge horizon over the world. From the author analysis figure in this study, it can be noted that out of 36 authors, only 12 researchers had a connection with each other. This shows the shortage of sharing research interests between authors and countries. Even though the number of publications has increased over the past 22 years, the collaboration between researchers in the ergonomics domain is still in the early stage. Besides the authors Li, Seo, Lee, and Yu, others have moderate cooperation. Aside from them, authors from the USA had the most connections with other countries. This area of research needs comprehensive consideration, encouragement, and collaboration from more academics worldwide, and across several fields. 

#### 4.2.3. Limitations of Existing Risk Assessment Methods

The several studies implemented in the construction sector to assess the risk involved in various activities encounter some limitations in practical scenarios. Some studies used questionnaires as a technique to identify the prevalence of WMSD risk factors among workers by conducting telephonic or face-to-face interviews. However, this may cause an interruption during the work. Another method is adopting observational methods such as REBA, RULA, OWAS, and PATH. The major disadvantage of adopting this technique is that it requires an expert observer, and the results can be subjective. To overcome this, scientists have implemented various wearable sensor-based technologies including IMU to collect postural data. The intrusiveness and discomfort created while performing activities led to the use of computer–vision-based technology in risk assessment. Studies have been conducted by combining vision-based technologies and observational tools to identify unsafe postures. 

However, most of the studies evaluated their methods on laboratory experiments which vary significantly with onsite conditions. Hence, future researchers should validate their technique using field observations to obtain more accurate results. Similarly, the presence of occlusion in the captured videos or images is not considered which can result in the inaccurate risk analysis of workers. Several occlusion rectification studies have developed in other sectors that include the Bayesian approach and the use of temporal convolutional networks (TCN). The problem of occlusion can be overcome by incorporating these methods in the pose estimation framework in the construction sector. Furthermore, most of the state-of-the-art pose estimation frameworks like Open Pose require powerful hardware for inference, and applying methods like Mediapipe Pose can tackle this. Solving these problems in future studies can benefit construction workers’ accurate unsafe posture estimation in real time. 

## 5. Conclusions

This paper summarizes the statistical analysis and mapping of a bibliometric review of the recent advancements in the topic of ergonomic risk assessment in the construction industry. This analysis explored the general trends on keywords most often used in publications, journals that published most articles, countries with significant contributions, and the most active authors in this field. In total, 330 articles published between 2000 and 2022 obtained from the WoS database are scientifically analyzed using the software VOSviewer. The co-occurring keyword analysis indicates that WMSDs and the construction industry are the hot spots in this research area. The co-authorship analysis enabled us to discover the most productive and cited authors. Li had the highest number of documents with 511 citations, whereas Seo had the lowest number of publications with the second highest citations of 182. Moreover, the leading countries are determined by their contribution to construction risk assessment. The. USA and China have contributed 131 and 33 documents, respectively. The publications from the USA, China, and the Netherlands received the highest citations.

Although this study provides a decent understanding of the fundamental elements, knowledge structure, and evolution trends in the risk assessment domain, there are some limitations to be addressed in the future. Firstly, we collected literature from only one database which limited the scope of data that led to insufficient coverage of 330 articles. Incorporating other databases such as Scopus can broaden the knowledge domain of research interest around the globe. This study conducted a scientometric analysis that examined only the title, keyword, and abstract, and to get a more in-depth information content analysis can be conducted in the future. Furthermore, the analysis of the networks and the overview provided can be subjective to the author’s knowledge and the results can vary depending on the parameters chosen in the visualization software. Overall, the findings of this study may benefit scholars and practitioners in identifying and evaluating emerging trends and research areas associated with the risk assessment of construction workers.

## Figures and Tables

**Table 1:** Country analysis (descending order according to documents).

Country	Documents	Citations	Total Link Strength
USA	131	2987	21
China	33	785	13
Canada	30	489	12
Netherlands	24	640	5
Sweden	20	559	15
England	17	272	9
Denmark	14	410	8
South Korea	14	152	6
France	12	117	3
Norway	10	136	6

**Table 2:** Co-authorship analysis (descending order according to documents).

Author	Documents	Citations	Average Citations	Author	Documents	Citations	Average Citations
Li, Heng	14	511	37	Rempel, David	6	50	8
Dale, Ann Marie	9	156	17	Sinsel, Erik W.	6	21	4
Veiersted, Kaj Bo	9	135	15	Warren, Christopher M.	6	21	4
Dai, Fei	7	39	6	Welch, Laura	6	95	16
Haas, Carl T.	7	104	15	Yu, Yantao	6	160	27
Knardahl, Stein	7	111	16	Abdel-Rahman, Eihab	5	47	9
Koch, Markus	7	118	17	Al-Hussein, Mohamed	5	97	19
Lunde, Lars-Kristian	7	118	17	Antwi-Afari, Maxwell Fordjour	5	79	16
Umer, Waleed	7	110	16	Descatha, Alexis	5	83	17
Wu, John Z.	7	39	6	Frings-Dresen, Monique H. W.	5	134	27
Barr, Alan	6	50	8	Han, Sanghyeok	5	92	18
Breloff, Scott P.	6	21	4	Kim, Sunwook	5	108	22
Buchholz, Bryan	6	103	17	Obonyo, Esther	5	29	6
Dutta, Amrita	6	21	4	Roquelaure, Yves	5	71	14
Hess, Jennifer A.	6	62	10	Ryu, Juhyeong	5	51	10
Lee, Sanghyun	6	174	2	Seo, Joonoh	5	182	36
Li, Xinming	6	47	8	Sluiter, Judith K.	5	125	25
Nussbaum, Maury A.	6	124	21	Zhao, Junqi	5	29	6

**Table 3:** List of journals (descending order according to documents).

Source	Documents	Citations	Impact Factor
*Automation in construction*	26	733	10.517
*Applied ergonomics*	24	478	3.94
*Journal of construction engineering and management*	21	336	5.292
*American journal of industrial medicine*	20	678	3.079
*Ergonomics*	19	478	2.561
*Scandinavian journal of work environment & health*	14	539	5.492
*Occupational and environmental medicine*	11	646	4.961
*BMC public health*	9	175	4.135
*International journal of industrial ergonomics*	9	124	2.884
*International journal of environmental research and public health*	8	65	4.614
*Safety science*	8	102	6.392
*Advanced engineering informatics*	7	167	7.862
*International archives of occupational and environmental health*	5	52	2.851
*Journal of occupational and environmental hygiene*	5	113	3.359
*Journal of occupational and environmental medicine*	5	167	2.306
*Occupational medicine-oxford*	5	74	5.629

## Data Availability

All relevant data are within the manuscript.
